# Effects of Schizophrenia, Bipolar Disorder, and Depression on Cardiopulmonary and Abdominal Organ Structure

**DOI:** 10.1016/j.bpsgos.2026.100783

**Published:** 2026-06-29

**Authors:** Shengsi Chu, Francesco Casanova, Renu Bala, Oliver D. Howes, Emanuele Osimo, Antonio de Marvao, Maddalena Ardissino, Declan P. O’Regan, Andrew McIntosh, Rona J. Strawbridge, Toby Pillinger, Jess Tyrrell

**Affiliations:** a Department of Clinical and Biomedical Sciences, University of Exeter, Exeter, United Kingdom; b Department of Psychosis Studies, Institute of Psychiatry, Psychology and Neuroscience, King’s College London, London, United Kingdom; c Department of Psychiatry, University of Cambridge, Cambridgeshire and Peterborough National Health Service Foundation Trust, Cambridge, United Kingdom; d King’s British Heart Foundation Centre of Research Excellence, School of Cardiovascular and Metabolic Medicine and Sciences, King’s College London, London, United Kingdom; e Department of Women and Children’s Health, King’s College London, London, United Kingdom; f Medical Research Council Laboratory of Medical Sciences, London, United Kingdom; g Institute of Clinical Sciences, Imperial College London, London, United Kingdom; h Centre for Clinical Brain Sciences, University of Edinburgh, Edinburgh, United Kingdom; i School of Health and Wellbeing, University of Glasgow, Glasgow, United Kingdom; j South London and Maudsley National Health Service Foundation Trust, London, United Kingdom

**Keywords:** Bipolar disorder, Genetics, Major depression, MRI imaging, Schizophrenia

## Abstract

**Background:**

People with severe mental illness (SMI) have shorter life expectancy, largely driven by physical health conditions. While lifestyle and psychotropic side effects contribute, peripheral organ dysregulation is intrinsic to SMI. Clarifying these effects could reveal novel therapeutic targets.

**Methods:**

Mendelian randomization (MR) was used to test the causal effects of genetic liability to schizophrenia, bipolar disorder, and major depressive disorder (MDD) on magnetic resonance imaging (MRI)–derived measures of peripheral organ structure and composition. Multivariable MR assessed lifestyle and metabolic mediators. One-sample MR and observational analyses in the UK Biobank (UKB) explored sex-specific effects. Two-sample MR used the largest genome-wide association study (GWAS) for schizophrenia (*N* = 175,799), bipolar disorder (*N* = 2,954,535), and MDD (*N* = 5,053,033). MRI-derived GWASs included up to 38,923 participants.

**Results:**

Genetic liability to all 3 SMIs associated with reduced peak diastolic strain rates, indicating impaired myocardial relaxation. Schizophrenia liability associated with smaller ventricular volumes, larger lung volumes, and higher liver iron levels. Bipolar disorder liability associated with lower right-sided cardiac volumes; higher left ventricular mass-to-volume ratio; and increased visceral, subcutaneous, and organ fat. MDD liability predominantly associated with greater abdominal and organ fat. Associations persisted after adjustment for body mass index, inflammation, insulin resistance, and smoking. One-sample MR in the UKB was directionally consistent and suggested sex-specific effects.

**Conclusions:**

SMI genetic liability exerts both shared and disorder-specific causal effects on peripheral organ structure, with myocardial stiffening (indicating poor cardiovascular prognosis) common to all, cardiopulmonary changes in schizophrenia, adipose-organ changes in MDD, and an intermediate phenotype in bipolar disorder. These cardiometabolic alterations support integrated screening and prevention strategies targeting cardiovascular and metabolic risk in SMI.

## Plain Language Summary

People with severe mental illness (such as schizophrenia, bipolar disorder, and depression) often die earlier, mainly due to physical health problems. This study used genetic methods to test whether these conditions directly affect body organs. We found that all 3 conditions are linked to reduced heart function, suggesting stiffer heart muscle. Schizophrenia mainly affected the heart and lungs, while bipolar disorder and depression were linked to increased body and organ fat. These findings suggest that physical health monitoring and early prevention should be a routine part of care for people with severe mental illness.

People with severe mental illness (SMI)—defined here as schizophrenia, bipolar disorder, and major depressive disorder (MDD)—have a life expectancy up to 20 years shorter than that of people without SMI (1, 2, 3). Most of this premature mortality is due to physical health conditions (4,5). Poor physical health in SMI is often attributed to the secondary effects of psychiatric illness, e.g., negative/depressive symptoms leading to a sedentary lifestyle (6), physical health side effects of psychotropics (7, 8, 9, 10), and inequitable health care access (11). However, metabolic and immune alterations are present at SMI onset (12, 13, 14); this suggests that dysregulation of organ systems outside the central nervous system is intrinsic to SMI (15). Elucidating these effects will improve understanding of SMI pathophysiology and may help identify novel therapeutic targets.

Cardiac disease is a major cause of excess mortality in people with SMI (16). Mendelian randomization (MR) studies have supported a causal relationship between some psychiatric conditions and cardiac disease risk, but not all. For example, genetically predicted schizophrenia and depression, but not bipolar disorder, associate with increased heart failure risk (17, 18, 19). Furthermore, genetically predicted depression, but not schizophrenia or bipolar disorder, is associated with increased coronary artery disease (CAD) risk (17,20,21). Some associations exhibit sex-specific effects, with genetic predisposition to depression conferring a greater risk of CAD in females (22). High polygenic risk scores (PRSs) for schizophrenia associated with more marked cardiac structural and functional variation as assessed using magnetic resonance imaging (MRI) (23). These alterations include reduced peak diastolic strain rates (PDSRs), indicating myocardial stiffness and diastolic dysfunction, which predict adverse cardiac outcomes and mortality (24,25). While PRS studies link schizophrenia to structural cardiac changes that worsen cardiac outcomes, they cannot establish causality. It is also unclear if similar effects occur in bipolar disorder and depression and if SMIs influence structural variations of other peripheral organs or body composition. Depression is causally associated with high body mass index (BMI) (26), while schizophrenia is linked to lower BMI (27); however, no previous studies have examined the causal effects of SMI on peripheral organ fat and abdominal fat volumes. Finally, the extent to which any causal effects of SMI on peripheral anatomical variation are mediated by lifestyle, metabolic, or sex-specific mechanisms is unknown. Metabolic and immune disturbance may be intrinsic to SMI, with insulin resistance and a proinflammatory state observed even in antipsychotic-naïve people with first-episode psychosis (15,28); such abnormalities may affect cardiopulmonary and abdominal organ structure. Smoking, which is more common among individuals with SMI (29), further contributes to systemic organ dysfunction. These pathways may mediate the effects of SMI on peripheral anatomy.

MR leverages the random allocation of genetic variants at conception to assess whether exposure has a causal influence on the outcome. As genetic variants are fixed prior to disease onset, MR can approximate a randomized trial, mitigating confounding and reverse causation. Here, we used 2-sample MR, with the largest available genome-wide association studies (GWASs), to test the causal association effects of schizophrenia, bipolar disorder, and MDD on the structure (e.g., organ volumes) and composition (e.g., fat content) of key cardiometabolic organs (e.g., heart, liver, and pancreas), as well as abdominal adiposity. Then, we used multivariate MR to assess whether these effects were mediated by BMI, insulin resistance, inflammation, and smoking. Finally, we analyzed individual-level data from UK Biobank (UKB), applying 1-sample MR to examine sex-specific associations.

## Methods and Materials

### Study Sample

#### Summary Statistics

To assess causal associations between SMI and cardiac structure and function and abdominal traits, we performed 2-sample MR using the most up-to-date European ancestry GWAS summary statistics. For SMI, we used summary statistics for schizophrenia from Trubetskoy *et al.* (30), MDD from Adams *et al.* (31), and bipolar disorder from O’Connell *et al.* (32).

Published GWAS summary statistics were used for 16 MRI-derived phenotypes for cardiopulmonary traits and 10 abdominal MRI measurements from UKB (33, 34, 35, 36, 37). A full list of GWAS datasets is provided in the Supplement.

#### UK Biobank

UKB is a cohort study of over 500,000 U.K. adults ages between 40 and 70 years at recruitment (2006–2010). The data include demographics, biomarkers, genetics, and linked medical records (38). Subsequently, a subset of individuals has undertaken an additional MRI visit (current data from 2014 to 2023).

For observational and 1-sample MR analyses we coded schizophrenia, bipolar disorder, and MDD using hospital inpatient records, primary care data, and death records (see the Supplement for diagnostic codes). Protocols for standardized cardiovascular and abdominal MRI acquisition have been detailed previously (35,39). Twenty cardiopulmonary phenotypes reflecting ventricular volumetry, systolic function, myocardial strain, concentricity, and lung volume were selected (23). Where available, we used cardiac phenotypes indexed to body surface area to normalize for body size and enhance comparability between individuals. These indexed measures were available only in the UKB individual data. MRI-derived measures of peripheral organ structure, composition, and adiposity were also considered (Supplement).

### Statistical Analyses

All analyses were performed in R (version 4.5.1).

#### Two-Sample MR

We performed 2-sample MR using the largest available GWAS data for SMIs and cardiopulmonary/abdominal MRI. Genetic variants (or single nucleotide polymorphisms [SNPs]) were included as exposures if they reached genome-wide significance (GWS) (*p* < 5 × 10^−8^) in the primary GWAS (Tables S1–S3). Instrument strength was assessed via minimum, maximum, and mean *F* statistics (Table S4). Potential pleiotropy of each variant was assessed using the GWAS catalog (https://www.ebi.ac.uk/gwas/home) (Tables S1–S3), and if an exposure variant was unavailable in the outcome GWAS, suitable proxies (within 500 kb and *r*^2^ > 0.8) were identified using LDProxy (https://ldlink.nih.gov/ldproxy) (Tables S1–S3).

Exposure SNPs and proxies were extracted from the outcome GWAS, representing the association of the outcome and exposure-trait-SNP. Published coefficients from the primary GWAS (Tables S1–S3) represent the exposure association with the exposure-trait-SNP. A custom pipeline performed 4 2-sample MR methods: inverse-variance weighting (IVW), MR-Egger (40), weighted median (WM), and penalized weighted median (PWM) (41). IVW represents our main analyses, with MR-Egger, WM, and PWM used as sensitivity analyses to account for unidentified pleiotropy (42). The methods are described in the Supplement.

When using SMI summary statistics as exposures, causal estimates for the outcomes were converted to represent the per-unit difference in outcome per doubling of genetic liability to binary exposure (43). All βs and SEs were multiplied by 0.693 (log_e_2) to convert the outcome to a doubling in the genetic liability of the exposure.

To assess and account for pleiotropy, we removed variants that reached GWS with the outcome and with known associations with related traits (Tables S1–S3).

#### MRlap: Account for Potential Bias Including Weak Instruments in Reverse MR

The MDD and bipolar disorder GWASs include a small number of individuals from the UKB, creating modest sample overlap between the exposure and outcome datasets. Such overlap can induce winner’s curse, inflating SNP-exposure estimates and biasing causal estimates toward the null or observational direction. Furthermore, to test for reverse causality, a relaxed *p*-value threshold was required (1 × 10^−5^ for cardiopulmonary and 1 × 10^−6^ for abdominal organs/adiposity MRI), increasing the risk of weak instrument bias. To address both sources of bias, we used MRlap (44). This method corrects for weak instrument bias and winner’s curse, while accounting for sample overlap and its effect as a modifier of these biases. Detailed information can be found in Mounier *et al.* (44) and the Supplement.

#### Multivariable MR

To disentangle the direct effects of SMIs from mediation by metabolic, inflammatory, or behavioral traits, we performed multivariable MR (MVMR) (45) adjusting for BMI (46), insulin resistance (total triglyceride: high density lipoprotein–cholesterol ratio [TG:HDL]) (47), C-reactive protein (CRP) (48), and smoking (cigarettes per day) (49). MVMR-Horse, a Bayesian approach (50), was additionally applied to validate the findings from standard MVMR. For more information, see the Supplement.

#### UKB Observational Analyses

We tested observational associations between SMI and MRI-derived phenotypes using robust multivariable linear or logistic regression, adjusting for age, sex, imaging center, BMI, smoking status (current/former/never), and Townsend deprivation index in up to 38,785 individuals of all ancestries and 36,769 individuals of European ancestry.

#### One-Sample MR

Individual-level UKB data were used to perform 1-sample MR and stratified analyses. We used SMI genetic risk scores as the exposures and tested associations with MRI phenotypes. Univariate MR analyses were performed using a 2-stage least squares approach for continuous outcomes and the 2-stage predictor substitution for binary outcomes. Models were adjusted for the first 5 genetic principal components and only included unrelated individuals of European ancestry to reduce confounding due to population stratification. Analyses were performed in all individuals, and men and women separately to test for sex-specific effects, and in a subset of nonsmokers to account for potential confounding by smoking.

#### Multiple Testing Correction

To account for multiple testing, we applied a Benjamini-Hochberg (BH) false discovery rate (FDR) correction, which is well suited to analyses involving correlated exposures and outcomes as it remains valid under common dependency structures while maintaining statistical power by controlling the expected proportion of false discoveries rather than overpenalizing multiple related tests. FDR correction was applied separately within each SMI, reflecting the disorder model–specific nature of these analyses. Associations with FDR-adjusted *p* values < .05 were considered statistically significant, with nominal associations only reaching *p* < .05 in non–FDR-adjusted models.

## Results

### Two-Sample MR: Schizophrenia and Cardiopulmonary Traits

**Figure 1:**
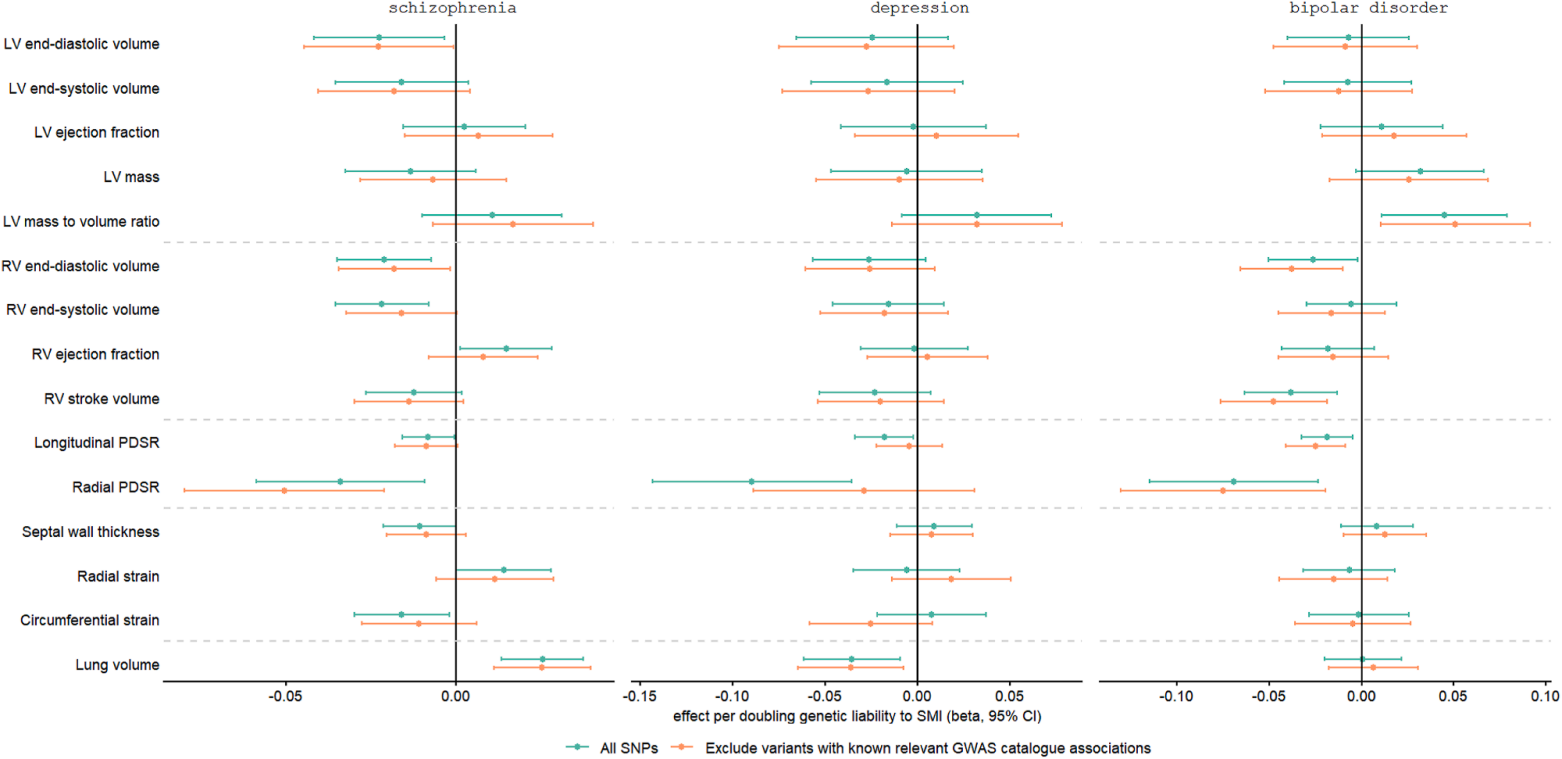
Causal effects of SMI genetic liability on cardiopulmonary traits. The causal effects of genetic liability to SMI on cardiopulmonary traits from IVW estimator and excluding SNPs with known GWAS catalog mental health, cardiometabolic, and immunologic associations. β coefficients and SEs were transformed to reflect effects per doubling of genetic liability to SMI. IVW estimations including all SNPs are represented in green, and estimations excluding SNPs with known GWAS catalog mental health, cardiometabolic, and immunologic associations are represented in orange. Error bars represent 95% CIs of the effect sizes. GWAS, genome-wide association study; IVW, inverse-variance weighting; LV, left ventricular; PDSR, peak diastolic strain rate; RV, right ventricular; SMI, severe mental illness; SNP, single nucleotide polymorphism.

Higher schizophrenia genetic liability was associated with variation across several cardiopulmonary traits (8/15 outcomes, 4 surviving BH-FDR correction) (Figure 1; Table 1; Table S5). A doubling in schizophrenia genetic liability was associated with lower longitudinal (−0.008, 95% CI [−0.016 to −0.001]) and radial (−0.038, 95% CI [−0.050 to −0.009]) PDSR, smaller right ventricular end-diastolic volume (RVEDV), left ventricular end-diastolic volume (LVEDV), and right ventricular end-systolic volume (RVESV) values (RVEDV = −0.021, 95% CI [− 0.035 to −0.007]; LVEDV = −0.023, 95% CI [−0.042 to −0.004]; RVESV = −0.022, 95% CI [−0.036 to −0.008]). In contrast, a doubling in schizophrenia genetic liability was associated with larger lung volumes (0.025, 95% CI [0.013 to 0.037]). Findings were generally consistent after excluding known pleiotropic loci and using pleiotropy-robust methods, although confidence intervals were wider (Table S5). MR-Egger provided no evidence of horizontal pleiotropy (*p*_MR-Egger intercept_ > .05) (Table S5). There was tentative evidence that schizophrenia was associated with higher right ventricular ejection fraction (RVEF) (0.015, 95% CI [0.001 to 0.028]) and lower mean peak circumferential strain (−0.016, 95% CI [−0.030 to −0.002]), although findings were less consistent across pleiotropy-robust methods and MRlap analyses (Tables S5 and S6). There was no evidence that schizophrenia genetic liability was associated with variations in left ventricular mass (LVM), left ventricular mass to end-diastolic volume ratio (LVMVR), left ventricular ejection fraction (LVEF), right ventricular stroke volume (RVSV), mean wall thickness or mean peak radial strain (Tables S5 and S6).

There was no evidence for reverse causality (Figure S1), with none of the cardiopulmonary traits associating with schizophrenia (Table S6).

MVMR analyses did not materially alter the overall pattern of results (Figure S2). Associations with radial strain emerged across adjustments (except CRP where it did not survive FDR correction), whereas those for longitudinal PDSR and LVEDV were attenuated by CRP and smoking, respectively (Figure S2 and Table S7).

### UKB: Schizophrenia and Cardiopulmonary Traits

Observational analysis of schizophrenia in UKB was underpowered, but suggested associations with lower RVEF and higher longitudinal strain (Table S8). One-sample MR produced consistent results with 2 samples. A doubling in schizophrenia genetic liability associated with 13 of 20 cardiopulmonary traits, with 9 surviving BH-FDR correction (Table S9). Higher schizophrenia genetic liability associated with lower indexed-LVEDV (LVEDVi), LVESVi, RVEDVi, RVESVi, higher RVEF, and larger lung volumes (Figure S3 and Table S9). Results were consistent in men and women (Figure S3 and Table S9), although the reduction in LVESVi and RVESVi and the increase in radial strain was nominally greater in men; however, none of these differences survived FDR correction. Stratified MR in nonsmokers was consistent with the overall MR results (Table S9).

### Two-Sample MR for Bipolar Disorder and Cardiopulmonary Traits

Higher bipolar disorder genetic liability associated with multiple cardiopulmonary traits (5/15 outcomes, 4 surviving BH-FDR correction) (Figure 1 and Table 1). A doubling in genetic liability for bipolar disorder associated with lower longitudinal (−0.019, 95% CI [−0.033 to −0.005]) and radial (−0.069, 95% CI [−0.115 to −0.023]) PDSR (Figure 1 and Table 1), lower RVSV (−0.038, 95% CI [−0.063 to −0.013]), nominally lower RVEDV (−0.026, 95% CI [−0.051 to −0.002]) and higher LVMVR (0.048, 95% CI [0.013 to 0.083]). Associations remained consistent after excluding pleiotropic variants and when using pleiotropy-robust MR methods, including MR-Egger where there was no evidence of horizontal pleiotropy (Tables S5 and S6). There was no consistent evidence for bipolar disorder causing changes in RVEF, LVEF, LVM, RVESV, left ventricular end-systolic volume (LVESV), LVEDV, and lung volume. There was limited evidence of reverse causality, with higher genetically instrumented lung volume, and mean peak radial strain associated with higher odds of bipolar disorder, although these did not survive FDR correction (Table S6).

MVMR analyses yielded broadly similar results (Figure S2 and Table S10). However, controlling for smoking and insulin resistance attenuated most associations. The association with RVEDV was no longer present after the adjustments. Furthermore, an association emerged between bipolar disorder and higher LVM when accounting for smoking and CRP (Table S10), which was not previously observed.

### UKB: Bipolar Disorder and Cardiopulmonary Traits

In the UKB, observational analyses were underpowered and showed only nominal associations with lower LVEDV (Table S8). One-sample MR analyses indicated a doubling in bipolar disorder genetic liability associated with lower LVEDV, LVESV, and LVESVi and higher LV radial strain in nonsmokers only (Table S9). There were no clear differences observed between men and women (Table S9).

### Two-Sample MR: MDD and Cardiopulmonary Traits

Higher genetic liability for MDD was associated with 3 of 15 cardiopulmonary traits (2 survived BH-FDR correction) (Figure 1; Table 1; Table S5). A doubling in MDD genetic liability was associated with lower radial (−0.090, 95% CI [−0.143 to −0.036]), longitudinal PDSR (−0.018, 95% CI [−0.034 to −0.002]), and smaller lung (−0.036, 95% CI [−0.062 to −0.009]) volume. Associations were directionally consistent across sensitivity analyses using pleiotropy-robust methods, with no MR-Egger evidence of horizontal pleiotropy and after excluding known pleiotropic loci (Tables S5 and S6), but they had larger uncertainty. There was no evidence of a causal effect of MDD on other cardiopulmonary traits (Tables S5 and S6).

There was no evidence of reverse causality, with none of the cardiopulmonary measures associated with MDD after accounting for multiple testing (Table S6). MVMR did not materially alter findings, except that the association with lung volume was attenuated (except for CRP) (Figure S2 and Table S11).

### UKB: MDD and Cardiopulmonary Traits

Observational analyses in the UKB suggested that depression (2147 cases in all ancestries and 2038 in European ancestry) associated with lower indexed biventricular volumetric measures, LVEF, and LVMi and nominally decreased radial strain and lung volume (Table S8). Individual-level MR analyses were consistent with 2-sample MR, providing limited causal evidence for the role of MDD on cardiopulmonary outcomes. There was tentative evidence that higher MDD genetic liability associated with lower lung volume and lower LVEF (Figure S3 and Table S9), but these did not survive FDR correction. In men, there was nominal evidence that higher MDD genetic liability associated with thicker myocardial walls, whereas no association was observed in women (*p*_difference_ = .019).

### Two-Sample MR: Schizophrenia and Abdominal Traits

**Figure 2:**
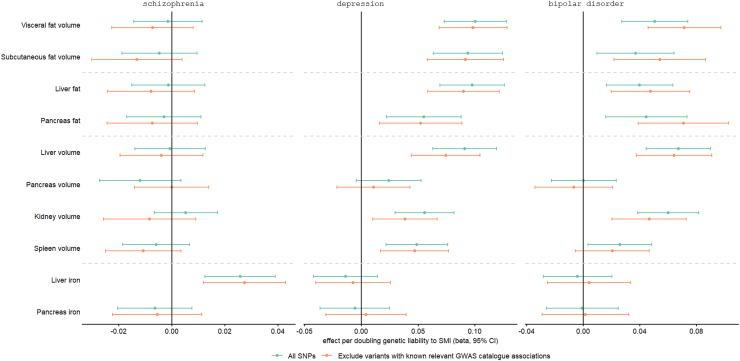
Causal effects of SMI genetic liability on abdominal and body composition traits. The causal effects on abdominal and body composition traits from IVW estimator and excluding SNPs with known GWAS catalog mental health and cardiometabolic associations. β coefficients and SEs were transformed to reflect effects per doubling of genetic liability to SMI. IVW estimations including all SNPs are represented in green, and estimations excluding SNPs with known GWAS catalog associations are represented in orange. Error bars represent 95% CIs of the effect sizes. GWAS, genome-wide association study; IVW, inverse-variance weighting; SMI, severe mental illness; SNP, single nucleotide polymorphism.

Higher schizophrenia genetic liability associated with limited variation in abdominal organ traits, including measures of adiposity (Figure 2; Table 2; Table S12). A doubling in schizophrenia genetic liability only associated with higher liver iron level (0.026, 95% CI [0.012 to 0.039]), which remained consistent with more pleiotropy-robust methods, removing known pleiotropic loci and after accounting for sample overlap (Tables S12 and S13). No consistent associations were observed for the other abdominal traits (Tables S12 and S13).

There was nominal evidence of a bidirectional association between schizophrenia and liver iron, with higher genetically predicted liver iron level associated with higher schizophrenia odds (Figure S4 and Table S13), but this did not survive FDR correction. MVMR analyses did not alter our findings, although adjustment for CRP attenuated the liver iron association (Figure S5 and Table S14).

### UKB: Schizophrenia and Abdominal Traits

In observational analyses, schizophrenia was nominally associated with higher subcutaneous fat volume (Table S15). In contrast, one-sample MR analyses were consistent with the 2-sample MR, identifying an association only between higher schizophrenia genetic liability and liver iron levels (Figure S6 and Table S16).

### Two-Sample MR: MDD and Abdominal Traits

MR provided evidence that higher MDD genetic liability was associated with 7 of 10 abdominal MRI traits (all survived FDR correction). A doubling in MDD genetic liability was robustly associated with greater visceral and subcutaneous adipose tissue volumes (β = 0.101, 95% CI [0.073 to 0.128] and β = 0.094, 95% CI [0.064 to 0.124], respectively) (Figure 2; Table 2; Table S12), higher liver fat (0.098, 95% CI [0.069 to 0.126]), larger liver (0.091, 95% CI [0.063 to 0.119]), spleen (0.049, 95% CI [0.022 to 0.076]), and kidney volumes (0.056, 95% CI [0.030 to 0.082]). Associations were consistent when excluding pleiotropic loci and using pleiotropy-robust MR methods, with no MR-Egger evidence of horizontal pleiotropy (Figure 2; Tables S12 and S13). There was no evidence of an association between MDD genetic liability and pancreas volume and liver and pancreas iron levels (Tables S12 and S13).

MVMR did not alter our findings, except that the association with spleen volume was attenuated adjusting for insulin resistance (Figure S5 and Table S17). There was limited evidence of reverse causality, with only higher saturated fat volume associating with higher MDD odds (Figure S5 and Table S13).

### UKB: MDD and Abdominal Traits

In the UKB, MDD robustly associated with many abdominal traits (Table S15). For example, MDD associated with higher subcutaneous and visceral fat volumes and pancreas volumes (Table S15). One-sample MR confirmed these findings, with further stratified analyses demonstrating that the associations with visceral and subcutaneous fat volumes were nominally stronger in men (*p*_difference visceral_ = .019), whereas the association with pancreas volume was only present in women (Figure S6 and Table S16).

### Two-Sample MR: Bipolar Disorder and Abdominal Traits

Higher bipolar disorder genetic liability associated with 7 of 10 abdominal MRI traits (all survived BH-FDR correction) (Figure 2 and Table 2). A doubling in bipolar disorder genetic liability associated with higher visceral (0.051, 95% CI [0.027 to 0.074]) and subcutaneous (0.037, 95% CI [0.010 to 0.064]) adipose tissue volume, larger liver volume (0.067, 95% CI [0.045 to 0.090]), higher liver fat (0.040, 95% CI [0.016 to 0.063]), higher kidney volume (0.060, 95% CI [0.039 to 0.082]) and higher pancreas fat (0.045, 95% CI [0.016 to 0.073]), and larger spleen volume (0.026, 95% CI [0.003 to 0.049]). There was no evidence for association between bipolar disorder and liver or pancreas iron levels or pancreas volume. Findings were generally consistent after excluding pleiotropic loci, using pleiotropy-robust methods, and accounting for sample overlap (Tables S12 and S13). Reverse MR analyses provided limited evidence of reverse causality (Figure S4 and Table S13), with only higher visceral adipose tissue associating with higher odds of bipolar disorder.

MVMR analyses did not substantially alter our results (Figure S5 and Table S18). However, the association with liver fat was attenuated after adjusting for insulin resistance and CRP, and the association with subcutaneous fat volume disappeared when controlling for smoking. When accounting for insulin resistance, bipolar disorder liability associated with higher pancreas volume (Table S18).

### UKB: Bipolar Disorder and Abdominal Traits

In the UKB, observational analyses were underpowered but showed nominal associations with higher kidney volume and lower pancreas volume (Table S15). One-sample MR showed higher bipolar disorder liability associated with higher visceral and subcutaneous fat volumes and pancreas fat (Figure S6 and Table S16).

## Discussion

**Figure 3:**
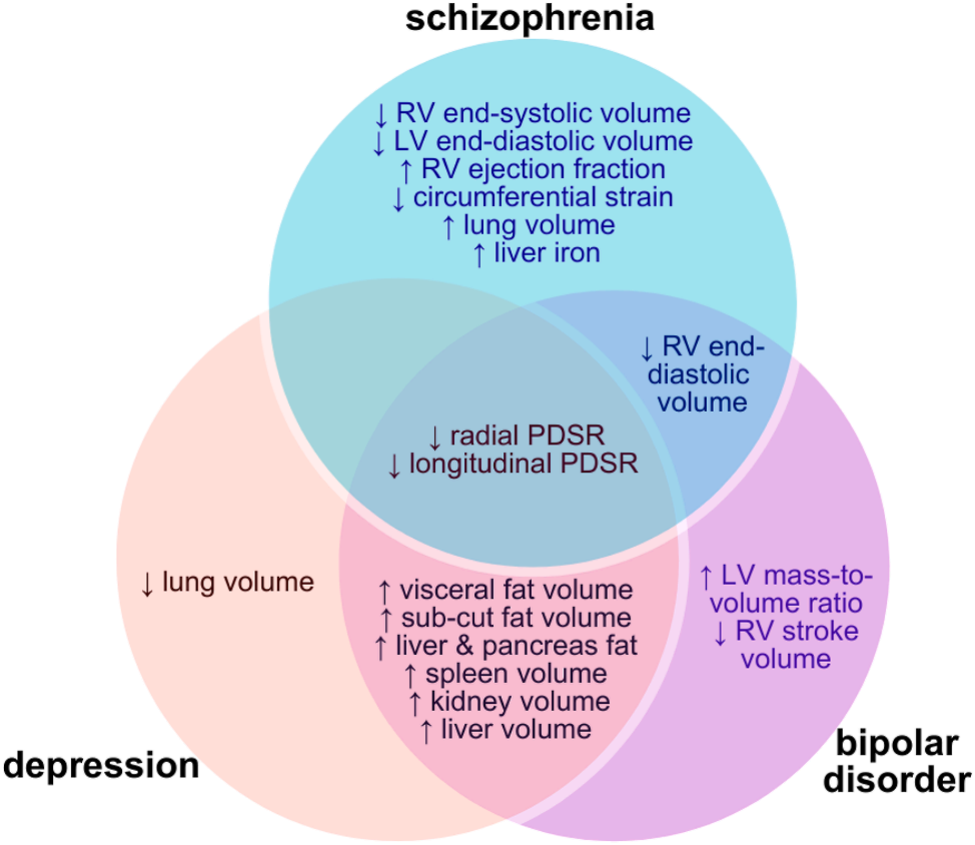
Shared and distinct causal effects of severe mental illness on peripheral anatomy and body composition. Summary of shared and distinct causal effects of schizophrenia, bipolar disorder, and depression on peripheral anatomy and body composition, based on 2-sample Mendelian randomization results. LV, left ventricular; PDSR, peak diastolic strain rate; RV, right ventricular; sub-cut, subcutaneous adipose tissue.

This study demonstrates both shared and distinct effects of SMI on cardiometabolic organ structure and body composition (Figure 3). Schizophrenia showed a predominantly cardiopulmonary phenotype, characterized by smaller left- and right-ventricular volumes, larger lung volumes, and higher liver iron levels. In contrast, bipolar disorder and depression showed a systemic adipose-organ phenotype, with strong associations with increased visceral and subcutaneous adiposity and larger abdominal organ volumes. Across disorders, PDSR was lower, indicating reduced cardiac elasticity. The cardiac signature of bipolar disorder overlapped partly with schizophrenia, with lower right-sided ventricular volumes, but was distinguished by a higher left ventricular mass-to-volume ratio, suggesting early concentric remodeling. In MDD, cardiopulmonary alterations were limited to reduced PDSR and smaller lung volumes. These findings indicate myocardial stiffening across SMI, with predominant cardiopulmonary changes in schizophrenia, metabolic-adipose changes in depression, and an intermediate form in bipolar disorder with changes in both cardiac structure and adiposity.

To our knowledge, this is the first study to comprehensively examine the causal effects of genetic proxies/instruments for SMI on cardiopulmonary and abdominal organ structure. Our findings extend prior work linking schizophrenia PRSs to reduced PDSR by demonstrating a shared causal effect across disorders (23). We add to evidence that genetic liability for mood disorders is an important determinant of adiposity, building on previous MR studies showing that depression associates with higher BMI (51). We found no association between adiposity and schizophrenia, confirming previous findings (52). However, both depression and bipolar disorder share evidence of a causal effect on increased abdominal fat volumes and organ fat content. These effects persisted after accounting for BMI and smoking, while adjustment for insulin resistance and CRP partially attenuated the association between bipolar disorder and liver fat, suggesting that metabolic and inflammatory pathways may partially mediate this effect. Our findings complement previous evidence showing that genetic liability to schizophrenia is associated with lower BMI (23), underscoring the contrast between the relative leanness linked to schizophrenia genetic risk and the adipose-organ phenotype observed in mood disorders. Sex-stratified analyses revealed several nominal differences in effect patterns, but these did not survive multiple testing and should be explored in more detail in larger studies. One-sample MR analyses in the UKB were broadly consistent with the 2-sample MR, and the available observational data showed concordant directions of effect, particularly reinforcing the depression-adiposity associations.

The association we observed between SMI and reduced PDSR (i.e., reduced cardiac elasticity) could arise through several underlying mechanisms, including immune pathways (e.g., inflammation and fibrosis) (53, 54, 55), metabolic dysfunction (e.g., insulin resistance) (56), and environmental exposures (e.g., smoking). Immune and metabolic abnormalities are well established in SMI (12, 13, 14); our previous study indicated that transforming growth factor β (a key driver of fibrosis) and inflammatory signaling pathways may link schizophrenia polygenic risk to cardiac phenotypic variation (23). However, in the present analyses, accounting for BMI, insulin resistance, CRP, and smoking in multivariable MR models did not materially alter the associations between SMI and lower PDSR, suggesting that the observed myocardial stiffness is not fully explained by metabolic, inflammatory, or lifestyle factors. However, CRP is a nonspecific marker of inflammation, and robust multivariable MR instruments for more specific inflammatory or fibrotic markers are not yet available. Future work should focus on identifying the causes of impaired myocardial relaxation in SMI. The persistence of the PDSR effect after adjustment supports the hypothesis that myocardial stiffening may represent a core, pleiotropic feature of SMI, warranting further mechanistic and therapeutic investigation.

The psychiatric GWASs used to derive instruments were based on clinically ascertained case populations; therefore, MR estimates may partly reflect downstream consequences of SMI, including pharmacological treatment exposure, alongside direct biological effects of genetic liability. This is relevant because antipsychotics have been implicated in cardiac structural alterations independent of metabolic effects (54). However, in our previous UKB PRS study, higher schizophrenia genetic liability in individuals without diagnosed schizophrenia was associated with smaller cardiac volumes and reduced PDSR, with findings unchanged after excluding participants prescribed antipsychotics (23). Consistency between these observations and the present MR findings argues against psychotropic exposure as the sole explanation for the observed multisystem associations.

The association between higher genetic liability to schizophrenia and larger lung volumes warrants cautious interpretation and further analysis. One possible explanation is poorer lung health in schizophrenia potentially driven by smoking, including a much higher risk of chronic obstructive pulmonary disease (57), which is characterized by air trapping and increased lung volumes. However, MVMR adjustment for smoking and stratified analysis within nonsmokers did not alter our findings, suggesting that smoking is unlikely to fully explain this association. Other plausible mechanisms include antipsychotic effects on respiratory function (58) or shared genetic architecture with lung function (59).

Most causal associations between genetic risk for depression or bipolar disorder and abdominal phenotypic variation can be explained by increased adiposity. For example, increased liver fat increases liver volume, and increased splenic volume may be due to increased portal pressures in the context of liver adiposity. The causal link between depression and adiposity has previously been examined, with a variety of environmental and biological pathways implicated (26). Our findings add to the evidence base of the importance of iron homeostasis in schizophrenia, with both iron deficiency and iron overload contributing to schizophrenia pathophysiology (30,60,61). Altered subcortical brain iron levels are observed in people with schizophrenia, and it is hypothesized that these alterations contribute to alterations in dopaminergic neurotransmission implicated in psychosis (62). Further work is required to determine if there is a shared pathophysiological pathway leading to alterations in both brain and liver iron levels. The finding of a causal effect for bipolar disorder, but not for schizophrenia or depression, on increased LVMVR (indicative of concentric remodeling that may be associated with pressure overload) may indicate unique pathways to cardiac dysfunction and disease in people with bipolar disorder, which should be explored in future studies.

A key finding is a common causal effect of SMI on reduced PDSR, indicating increased myocardial stiffness. Myocardial stiffness causes diastolic dysfunction, which predicts major cardiovascular events and all-cause mortality (24,25). Excess cardiac mortality is well documented in people with SMI, and novel approaches are required to address cardiac diseases in this cohort (16). Further work is required to determine if the SMI-PDSR causal pathway represents a novel therapeutic target, as well as a potential biomarker for monitoring or prognostic stratification. Cardiac disease screening is already poor in people with SMI and gold-standard treatments are often delayed (63); the implication that genetic risk for schizophrenia affects cardiac structure and function in a manner associated with increased cardiac disease risk supports the argument for regular cardiac screening in patients from illness onset, and initiatives to facilitate this process are encouraged (64). Given the strong causal evidence for adiposity-related effects in depression and bipolar disorder, regular assessment and proactive management of weight and broader metabolic risk should also be prioritized in these groups. Together, these findings highlight the need for integrated cardiometabolic care across the SMI spectrum. While this study benefits from the scale and granularity of contemporary genetic and imaging resources, several limitations should be acknowledged. The UKB is not population representative, with evidence of healthy volunteer and participation bias in optional components such as the Mental Health Questionnaire and MRI substudy, which may limit generalizability (65). Moreover, UKB participants are predominantly of European ancestry, as are the GWAS summary statistics used for the 2-sample MR analyses. This restricts the applicability of our findings to other ancestral groups, and future work in more diverse cohorts is essential. We were unable to perform formal replication as the 2-sample MRI GWAS data came from the UKB. However, we were able to perform observational analysis and stratified analyses by sex and smoking status. Reverse MR analyses were comparatively underpowered, owing to fewer strong instruments and the need for relaxed selection thresholds, and may therefore be susceptible to weak instrument bias even with MRlap, limiting firm conclusions regarding reverse causality. Finally, no MR method is without assumptions, and residual bias due to pleiotropy or measurement error cannot be fully excluded. Nonetheless, this study has several key strengths. We applied a broad suite of state-of-the-art MR sensitivity approaches to address potential biases such as winner’s curse, pleiotropy, and sample overlap, thereby strengthening causal inference (44). We leveraged the largest available GWAS datasets for schizophrenia, bipolar disorder, and MDD and confirmed findings in UKB individual-level data. Finally, our use of MVMR enabled exploration of potential mediating mechanisms, further enhancing the robustness and interpretability of the results. However, we acknowledge other potential mediators should be considered in the future including, physical inactivity, sleep, and social isolation. These were considered beyond the scope of this study.

### Conclusions

Genetic liability for schizophrenia, bipolar disorder, and depression is linked to a consistent pattern of structural and compositional variation across cardiopulmonary and abdominal organs. Given that some of these effects are associated with increased risk of major adverse cardiovascular events, these alterations could contribute to the excess cardiometabolic morbidity and mortality associated with SMI. Further work is required to determine how these causal associations relate to increased morbidity and mortality rates in people with SMI and whether they represent novel therapeutic targets.

**Table 1:** The Causal Effects of Genetic Liability to SMI on Cardiopulmonary Traits From IVW Estimator and Excluding SNPs With Known GWAS Catalog Mental Health, Cardiometabolic, and Immunologic Associations

Exposure	Outcome	All SNPs				Excluding Variants With Known Relevant GWAS Associations			
		β	SE	*p* Value	Adjusted *p* Value	β	SE	*p* Value	Adjusted *p* Value
Schizophrenia	LVEDV	−0.023	0.01	.021	.063	−0.027	0.012	.022	.164
	LVESV	−0.016	0.01	.11	.137	−0.024	0.012	.04	.207
	LVEF	0.002	0.009	.795	.795	0.01	0.011	.358	.556
	LVM	−0.013	0.01	.176	.203	−0.014	0.011	.228	.556
	LVMVR	0.011	0.01	.313	.335	0.014	0.012	.267	.251
	RVEDV	−0.021	0.007	.003	.016	−0.02	0.009	.022	.154
	RVESV	−0.022	0.007	.002	.015	−0.018	0.009	.034	.164
	RVEF	0.015	0.007	.034	.068	0.01	0.008	.259	.375
	RVSV	−0.012	0.007	.084	.114	−0.014	0.008	.092	.193
	Longitudinal PDSR	−0.008	0.004	.036	.068	−0.005	0.005	.261	.165
	Radial PDSR	−0.034	0.013	.008	.029	−0.044	0.015	.004	.007
	Wall thickness	−0.011	0.005	.053	.08	−0.011	0.006	.08	.241
	Mean peak circumferential strain	−0.016	0.007	.026	.065	−0.017	0.009	.064	.262
	Mean peak radial strain	0.014	0.007	.053	.08	0.015	0.009	.102	.262
	Lung volume	0.025	0.006	4.65 × 10^−5^	.001	0.036	0.01	.0006	.007
Depression	LVEDV	−0.025	0.021	.239	.356	−0.028	0.024	.252	.494
	LVESV	−0.017	0.021	.429	.503	−0.027	0.024	.262	.494
	LVEF	−0.002	0.02	.914	.914	0.01	0.023	.651	.717
	LVM	−0.006	0.021	.777	.847	−0.01	0.023	.669	.717
	LVMVR	0.032	0.021	.123	.21	0.032	0.023	.172	.494
	RVEDV	−0.026	0.016	.094	.19	−0.026	0.018	.149	.494
	RVESV	−0.016	0.015	.302	.401	−0.018	0.018	.306	.51
	RVEF	−0.002	0.015	.897	.914	0.005	0.017	.749	.749
	RVSV	−0.023	0.015	.133	.21	−0.02	0.017	.251	.494
	Longitudinal PDSR	−0.018	0.008	.027	.118	−0.004	0.009	.63	.717
	Radial PDSR	−0.089	0.027	.001	.009	−0.029	0.031	.342	.513
	Wall thickness	0.009	0.01	.377	.47	0.007	0.011	.515	.703
	Mean peak circumferential strain	0.007	0.015	.623	.686	−0.025	0.017	.134	.494
	Mean peak radial strain	−0.006	0.015	.682	.736	0.018	0.016	.263	.494
	Lung volume	−0.036	0.013	.008	.04	−0.036	0.015	.013	.202
Bipolar disorder	LVEDV	−0.007	0.017	.668	.77	−0.009	0.02	.657	.704
	LVESV	−0.008	0.018	.661	.77	−0.012	0.02	.544	.68
	LVEF	0.011	0.017	.521	.77	0.018	0.02	.377	.515
	LVM	0.032	0.018	.073	.183	0.026	0.022	.24	.473
	LVMVR	0.045	0.017	.01	.039	0.051	0.021	.015	.044
	RVEDV	−0.027	0.012	.033	.098	−0.038	0.014	.008	.032
	RVESV	−0.006	0.012	.652	.77	−0.016	0.015	.268	.473
	RVEF	−0.018	0.013	.153	.329	−0.015	0.015	.316	.473
	RVSV	−0.038	0.013	.003	.023	−0.048	0.015	.001	.019
	Longitudinal PDSR	−0.019	0.007	.009	.039	−0.025	0.008	.002	.019
	Radial PDSR	−0.069	0.023	.003	.023	−0.075	0.028	.008	.032
	Wall thickness	0.008	0.01	.4	.749	0.013	0.011	.267	.473
	Mean peak circumferential strain	−0.002	0.014	.91	.947	−0.005	0.016	.765	.765
	Mean peak radial strain	−0.007	0.013	.598	.77	−0.015	0.015	.309	.473
	Lung volume	0.001	0.011	.947	.947	0.006	0.012	.6	.693

β coefficients and SEs were transformed to reflect effects per doubling of genetic liability to SMI. *p* Values were adjusted using the Benjamini-Hochberg procedure for multiple testing correction.

GWAS, genome-wide association study; IVW, inverse-variance weighted; LVEDV, left ventricular end-diastolic volume; LVEF, left ventricular ejection fraction; LVESV, left ventricular end-systolic volume; LVM, left ventricular mass; LVMVR, left ventricular mass to end-diastolic volume ratio; PDSR, peak diastolic strain rate; RVEDV, right ventricular end-diastolic volume; RVEF, right ventricular ejection fraction; RVESV, right ventricular end-systolic volume; RVSV, right ventricular stroke volume; SMI, severe mental illness; SNP, single nucleotide polymorphism.

**Table 2:** The Causal Effects of Genetic Liability to SMI on Abdominal and Body Composition Traits From Inverse-Variance Weighted Estimator and Excluding SNPs With Known GWAS Catalog Mental Health, Cardiometabolic, and Immunologic Associations

Exposure	Outcome	All SNPs				Excluding Variants With Known Relevant GWAS Associations			
		β	SE	*p* Value	Adjusted *p* Value	β	SE	*p* Value	Adjusted *p* Value
Schizophrenia	Visceral fat volume	−0.001	0.007	.819	.915	−0.011	0.011	.348	.56
	Subcutaneous fat volume	−0.005	0.007	.516	.86	−0.019	0.013	.131	.447
	Liver fat	−0.001	0.007	.849	.915	−0.011	0.012	.347	.56
	Pancreas fat	−0.003	0.007	.671	.915	−0.011	0.013	.391	.56
	Liver volume	−0.001	0.007	.915	.915	−0.006	0.012	.618	.687
	Pancreas volume	−0.012	0.008	.126	.631	−0.012	0.013	.343	.56
	Kidney volume	0.005	0.006	.391	.783	−0.0001	0.01	.989	.989
	Spleen volume	−0.006	0.006	.355	.783	−0.016	0.01	.134	.447
	Liver iron	0.026	0.007	.0002	.002	0.039	0.011	.0006	.006
	Pancreas iron	−0.006	0.007	.367	.783	−0.008	0.012	.516	.645
Depression	Visceral fat volume	0.101	0.014	2.08 × 10^−12^	2.08 × 10^−11^	0.098	0.015	3.27 × 10^−10^	3.27 × 10^−9^
	Subcutaneous fat volume	0.094	0.015	2.36 × 10^−9^	5.9 × 10^−9^	0.092	0.017	1.5 × 10^−7^	5.0 × 10^−7^
	Liver fat	0.098	0.015	4.34 × 10^−11^	2.17 × 10^−10^	0.09	0.016	3.72 × 10^−8^	1.86 × 10^−7^
	Pancreas fat	0.055	0.017	.001	.002	0.052	0.019	.005	.008
	Liver volume	0.091	0.014	3.83 × 10^−10^	1.28 × 10^−9^	0.074	0.016	2.32 × 10^−6^	5.8 × 10^−6^
	Pancreas volume	0.024	0.015	.101	.126	0.011	0.016	.513	.642
	Kidney volume	0.056	0.013	3.07 × 10^−5^	6.14 × 10^−5^	0.038	0.014	.008	.012
	Spleen volume	0.049	0.014	.0005	.001	0.047	0.015	.002	.005
	Liver iron	−0.014	0.014	.329	.366	−0.007	0.017	.662	.735
	Pancreas iron	−0.006	0.016	.712	.712	0.004	0.018	.822	.822
Bipolar disorder	Visceral fat volume	0.051	0.012	2.61 × 10^−5^	.000087	0.072	0.013	1.23 × 10^−7^	1.23 × 10^−6^
	Subcutaneous fat volume	0.037	0.014	.008	.013	0.054	0.016	.001	.002
	Liver fat	0.04	0.012	.001	.002	0.048	0.014	.001	.002
	Pancreas fat	0.045	0.015	.003	.005	0.071	0.016	1.91 × 10^−5^	6.35 × 10^−5^
	Liver volume	0.067	0.012	1.61 × 10^−8^	1.61 × 10^−7^	0.064	0.014	3.65 × 10^−6^	1.82 × 10^−5^
	Pancreas volume	0.0004	0.012	.974	.974	−0.007	0.014	.642	.802
	Kidney volume	0.06	0.011	1.01 × 10^−7^	5.05 × 10^−7^	0.047	0.013	.001	.002
	Spleen volume	0.026	0.012	.025	.036	0.021	0.013	.124	.176
	Liver iron	−0.004	0.012	.748	.935	0.004	0.015	.778	.864
	Pancreas iron	−0.001	0.013	.955	.974	0.002	0.016	.92	.92

β coefficients and SEs were transformed to reflect effects per doubling of genetic liability to SMI. *p* Values were adjusted using the Benjamini-Hochberg procedure for multiple testing correction.

GWAS, genome-wide association study; SMI, severe mental illness; SNP, single nucleotide polymorphism.

## Supplementary Material

Tables S1–S19

Key Resources Table
