# Supplementary material for: Effects of Schizophrenia, Bipolar Disorder, and Depression on Cardiopulmonary and Abdominal Organ Structure
Source: Biol Psychiatry Glob Open Sci. 2026 Jun 29;6(5):100783. doi: 10.1016/j.bpsgos.2026.100783 (PMC13449780; doi:10.1016/j.bpsgos.2026.100783)
Supplement: Supplementary file 1 [file mmc1.pdf]

SUPPLEMENTARY INFORMATION

Effects of Schizophrenia, Bipolar Disorder, and Depression on Cardiopulmonary and Abdominal Organ Structure

Chu *et al.*

Content

Supplementary text

1. Sources of summary statistics
2. UK Biobank (UKB)
3. UKB-derived phenotypes
4. Mendelian Randomisation (MR) Assumptions
5. MR Analyses
6. Reference

Supplementary figures

1. Supplementary Figure 1 - Causal effects on cardiopulmonary traits from inverse-variance weighted (IVW) estimator and MRlap
2. Supplementary Figure 2 - Causal effects on cardiopulmonary traits from inverse-variance weighted (IVW) estimator and multivariable MR (MVMR) analyses controlling for body mass index (BMI), smoking (cigarette per day), C-reactive protein (CRP), and insulin resistance

3. Supplementary Figure 3 - Causal effects and sex-specific effects on cardiopulmonary traits from one-sample Mendelian Randomisation (MR)
4. Supplementary Figure 4 - Causal effects on abdominal and body composition traits from inverse-variance weighted (IVW) estimator and MRlap
5. Supplementary Figure 5 - Causal effects on abdominal and body composition traits from inverse-variance weighted (IVW) estimator and multivariable MR (MVMR) controlling for body mass index (BMI), smoking (cigarette per day), C-reactive protein (CRP), and insulin resistance
6. Supplementary Figure 6 - Causal effects and sex-specific effects on abdominal and body composition traits from one-sample Mendelian Randomisation (MR)

Supplementary text

Sources of summary statistics

Severe Mental Illness

- Schizophrenia: summary statistics from Trubetskoy et al., with 74,776 cases and 101,023 controls.[1] This GWAS doesn't include individuals from the UKB.
- Bipolar disorder: summary statistics from O'Connell et al., with 158,036 cases and 2.8 million controls.[2] UKB contributes: 1,454 cases and 58,113 controls.
- Major depression: summary statistics from Major Depressive Disorder Working Group of the Psychiatric Genomics Consortium, with over 500,000 depression cases and one million controls, excluding 23&Me data.[3] UKB contributes 3,328 cases and 5,638 controls.

MRI imaging measures

Cardio-pulmonary traits

We used the following published GWAS summary statistics for 16 cardiopulmonary phenotypes:

- Left ventricular end-diastolic volume (LVEDV), left ventricular end-systolic volume (LVESV), left ventricular stroke volume (LVSV), and left ventricular mass (LVM), alongside left ventricular ejection fraction (LVEF), and left ventricular mass to end-diastolic volume ratio (LVMVR) in 16,923 individuals from UKB.[4]
- Right ventricular end-diastolic volume (RVEDV), right ventricular end-systolic volume (RVESV), and right ventricular stroke volume (RVSV), alongside right ventricular ejection fraction (RVEF) in 29,506 individuals from UKB.[5]
- Mean wall thickness at end diastole, circumferential strain and radial strain were available in 47,549 individuals from the UKB.
- Longitudinal peak diastolic strain rate ($PDSR_{ll}$) and radial peak diastolic strain rates ($PDSR_{rr}$) in 38,923 individuals from UKB.[6]
- Lung volume from up to 38,881 individuals in the UKB.[7]

Abdominal and body composition traits

- Other MRI phenotypes considered included: a) kidney volume, b) pancreas volume, iron and fat-content, c) liver volume, iron and fat-content, d) spleen volume and e) abdominal subcutaneous adipose tissue and visceral fat volumes. GWAS summary statistics were used from up to 38,881 individuals in the UKB.[7]

UK Biobank (UKB)

UKB is a population health study containing genetic, imaging, and health data of over 500,000 individuals across the UK. Participants were recruited between 2006 and 2010, and 99.5% were aged between 40 and 70 years at the time of the recruitment. Demographic, questionnaire, biological and genotypic data were systematically collected and anonymised. Informed consent was obtained from all participants for the use of their data in research. The UKB data are available to any bona fide researcher following an application. UKB was approved by the Northwest Multi-Centre Research Ethics Committee. For full information on UKB please see Bycroft et al.[8] Data access of this study was approved under application 103356. No participants were involved in the design and conduct of this study and were not directly notified of the conclusions. However, results will be communicated and key findings from studies are published by UKB on their free-to-access website https://www.ukbiobank.ac.uk/learn-more-about-uk-biobank/news.

UKB-derived phenotypes

Demographic characteristics of all participants included in our study were summarised in ST19.

GP-derived MDD

MDD was defined following Fabbri et al[9] using linked GP records in UKB. Participants with a) at least two diagnostic codes for a depressive disorder at any time point and b) no diagnostic code for psychotic disorders, bipolar disorder, and substance abuse-related disorders were identified as MDD cases. In our study, this resulted in up to 1,671 cases and 12,799 controls in unrelated Europeans with non-missing covariates and MRI data.

GP severe depression

GP severe depression was defined as having two psychiatric referrals recorded in primary care data. In our study, this corresponded to a maximum of 315 cases and 12,779 controls among unrelated Europeans with non-missing covariates and MRI data.

Depressed ever

Defined by patients with self-reported depression through a mental health questionnaire completed by participants at baseline. In our study, this yielded up to 5070 cases and 12,438 controls in unrelated Europeans with available covariates and MRI measurements.

Schizophrenia

Schizophrenia was defined using ICD-10 codes F20 and F25 from linked Hospital Episode Statistics records. This resulted in 23 cases and 30,782 controls in unrelated Europeans with non-missing covariates and MRI data.

Bipolar disorder

Bipolar disorder was defined using ICD-10 codes F30.1-F30.4 and F31.0-F31.9 from linked Hospital Episode Statistics records. In our study, this comprised a maximum of 73 cases and 14,797 controls in unrelated Europeans with non-missing covariates and MRI measurements.

MRI-derived phenotypes

Twenty cardiopulmonary phenotypes and nine abdominal organ and body composition phenotypes were included in UKB observational and one-sample MR analyses. All MRI measurements were obtained from instance 2.

The cardiopulmonary phenotypes include:

- Left ventricular end-diastolic volume (LVEDV, data field 22421), end-systolic volume (LVESV, data field 22422), ejection fraction (LVEF, data field 22420), and body surface area (data field 22427).
- Alternative derivation of LVEDV (LVEDV-derived, data field 24100), LVESV (LVESV-derived, data field 24101), and LVEF (LVEF-derived, data field 24103); right ventricular end-diastolic volumes (RVEDV, data field 24106), end-systolic volumes (RVESV, data field 24107), and ejection fraction (RVEF, data field 24109); left ventricular mass (LVM, data field 24105), mean end-diastolic left ventricular myocardial wall thickness (data field 24140), and peak longitudinal and radial strain (data field 24181 and 24174). The acquisition of these cardiac phenotypes has been described previously by Bai *et al.*[11]
- Lung volume (data field 21084), derived as described by Liu *et al.*[12]
- Five cardiac phenotypes were indexed to body surface area, indexed-LVEDV (LVEDVi, derived from LVEDV-derived), indexed-LVESV (LVESVi, derived from LVESV-derived), indexed-LVM (LVMi), indexed-RVEDV (RVEDVi), and indexed-RVESV (RVESVi).

Abdominal and body composition include:

- Left (data field 21081) and right kidney volume (data field 21082), pancreas volume (data field 21087), iron (data field 21091), and fat-content (data field 21090); liver iron (data field 21089); spleen volume (data field 21083); and abdominal subcutaneous adipose tissue (data field 21086) and visceral fat volume (data field 21085). Details of the protocol for acquiring these abdominal phenotypes can be found in Liu *et al.*[12]

Observations with outcome measurements three Interquartile Ranges (IQRs) below the first quartile or above the third quartile were excluded, and observations with zero values were excluded. Exclusions were minimal, with the proportion of excluded data being under 1% for nearly all measurements. The number of exclusions for each phenotype can be found in ST9 and ST16.

Mendelian Randomisation (MR) Assumptions

MR relies on several assumptions:

1. Exposure SNPs robustly associate with the measured exposure. The F-statistic quantifies this and can be approximated by the ratio of the SNP-exposure association estimate, $\hat{\beta}$ and its standard error, $SE(\hat{\beta})$, squared (equation 1).[13]

   Equation 1

   $$F = \left(\frac{\hat{\beta}}{SE(\hat{\beta})}\right)^2$$

   F-statistics were calculated for each variant (see ST1-3), and mean F-statistics for each 2-sample MR analysis are provided in ST4.
2. Exposure SNPs are not associated with confounding factors that bias conventional epidemiological associations.
3. Exposure SNPs only associate with the outcome through the risk factor.

MR Analyses

MR Analyses used in this study included Inverse Variance Weighting (IVW), MR-Egger, Weighed Median (WM) and Penalised Weighed Median (PWM) methods.

IVW

IVW can be performed in both fixed-effects and random-effects models. Fixed-effect model assumes the absence of horizontal pleiotropy (no direct effect of genetic instruments on the

outcome) and homogeneity[14]. Random-effect model relaxes these assumptions, handling heterogeneous effects and only requires balanced pleiotropy (the averaged pleiotropic effects equal zero) and the independence between the strength of variant-exposure association and pleiotropic effects (InSIDE assumption).[14] Both models additionally require NOME (no measurement error - with first-order weights) and monotonicity. In two-sample MR, when all MR assumptions are met, no sample overlap is present, and all genetic instruments are strong, the IVW method provides consistent and unbiased causal estimates. Random-effects IVW was used in this study to account for heterogeneous estimates.

MR-Egger

MR-Egger relaxes the no-pleiotropy assumption and only requires InSIDE to be met.[15] It additionally assumes NOME and effect-increasing directions of allele orders. Therefore, MR-Egger can provide valid causal estimates when IVs violate the exclusion restriction assumption, and the p-values of the intercept represent the presence of directional pleiotropic effects.[16] However, estimates from MR-Egger are less precise, and the risk of type 1 errors is increased when InSIDE assumptions are violated.

Weighed Median and Penalised Weighed Median

Weighed median is more robust to invalid IVs, providing consistent causal estimates when at least 50% of total weighted IVs are valid.[17] This method does not depend on the homogeneity assumption, allowing for heterogeneity among genetic variants. However, weighted median produces biased results when invalid weighted IVs are directional. In this case, the penalised weighted median method can reduce the bias by downsizing extreme estimates, controlling large weights based on the chi-square distribution of weighted deviation from IVW estimates.[17] This helps mitigate the effect of potential outlying variants distorting weighted median estimates while maximally maintaining weights of valid IVs.

MRlap

MRlap is a method able to handle sample overlap, weak instrument bias and winner's curse[18]. It utilises cross-trait LD-score intercept to account for sample overlap, and the heritability and polygenicity to correct for errors from weak instrument bias and winner’s curse caused by variant selection[18]. MRlap conducts statistical tests on the difference of significance of IVW effects and corrected estimation. In this study, we adopted IVW estimates if there was no difference suggested; otherwise, the corrected effects were used.

Multivariable MR (MVMR) and MVMR-Horse

MVMR extends univariable MR by simultaneously modelling the causal effects of multiple, potentially correlated exposures on the outcome.[19] MVMR can control for measured confounders and pleiotropy, as well as estimate direct effects not through mediators. Valid inference requires genetic instruments influence the outcome only through the included exposures, and the instruments are conditionally sufficiently strong. We used MVMR package (https://github.com/WSpiller/MVMR) for primary analysis. In addition we used a new method called MVMR-Horse, which employs a Bayesian MVMR framework that additionally accounts for both uncorrelated and correlated pleiotropy.[20] It adopts a horseshoe shrinkage prior to model pleiotropy, permitting some variant-specific pleiotropic effects to be shrunk towards zero while allowing non-zero effects to escape excessive shrinkage. Posterior distributions were estimated using Markov chain Monte Carlo (MCMC) sampling and causal effects were reported as posterior means and 95% credible intervals. MVMR-Horse was implemented using R code provided by the authors (https://github.com/aj-grant/mrhorse). MVMR-Horse was only implemented when there was evidence of an association with the normal MVMR methodology to assess for any issues with pleiotropy.

Reference

1. Trubetskoy V, Pardinas AF, Qi T, et al. Mapping genomic loci implicates genes and synaptic biology in schizophrenia. *Nature*. Apr 2022;604(7906):502–508. doi:10.1038/s41586-022-04434-5
2. O'Connell KS, Koromina M, van der Veen T, et al. Genomics yields biological and phenotypic insights into bipolar disorder. *Nature*. 2025/01/22 2025;doi:10.1038/s41586-024-08468-9
3. Adams MJ, Streit F, Meng X, et al. Trans-ancestry genome-wide study of depression identifies 697 associations implicating cell types and pharmacotherapies. *Cell*. 2025;doi:10.1016/j.cell.2024.12.002
4. Aung N, Vargas JD, Yang C, et al. Genome-Wide Analysis of Left Ventricular Image-Derived Phenotypes Identifies Fourteen Loci Associated With Cardiac Morphogenesis and Heart Failure Development. *Circulation*. Oct 15 2019;140(16):1318–1330. doi:10.1161/CIRCULATIONAHA.119.041161
5. Aung N, Vargas JD, Yang C, et al. Genome-wide association analysis reveals insights into the genetic architecture of right ventricular structure and function. *Nature Genetics*. 2022/06/01 2022;54(6):783–791. doi:10.1038/s41588-022-01083-2
6. Thanaj M, Mielke J, McGurk KA, et al. Genetic and environmental determinants of diastolic heart function. *Nat Cardiovasc Res*. Apr 2022;1(4):361–371. doi:10.1038/s44161-022-00048-2
7. Liu Y, Basty N, Whitcher B, et al. Genetic architecture of 11 abdominal organ traits derived from abdominal MRI using deep learning. *bioRxiv*. 2020:2020.07.14.187070. doi:10.1101/2020.07.14.187070
8. Bycroft C, Freeman C, Petkova D, et al. The UK Biobank resource with deep phenotyping and genomic data. *Nature*. Oct 2018;562(7726):203–209. doi:10.1038/s41586-018-0579-z

9. Fabbri C, Hagenaars SP, John C, et al. Genetic and clinical characteristics of treatment-resistant depression using primary care records in two UK cohorts. *Mol Psychiatry*. Jul 2021;26(7):3363–3373. doi:10.1038/s41380-021-01062-9
10. Wigmore EM, Hafferty JD, Hall LS, et al. Genome-wide association study of antidepressant treatment resistance in a population-based cohort using health service prescription data and meta-analysis with GENDEP. *Pharmacogenomics J*. Apr 2020;20(2):329–341. doi:10.1038/s41397-019-0067-3
11. Bai W, Suzuki H, Huang J, et al. A population-based phenome-wide association study of cardiac and aortic structure and function. *Nat Med*. Oct 2020;26(10):1654–1662. doi:10.1038/s41591-020-1009-y
12. Liu Y, Basty N, Whitcher B, et al. Genetic architecture of 11 organ traits derived from abdominal MRI using deep learning. *Elife*. Jun 15 2021;10doi:10.7554/eLife.65554
13. Bowden J, Del Greco MF, Minelli C, et al. Improving the accuracy of two-sample summary-data Mendelian randomization: moving beyond the NOME assumption. *Int J Epidemiol*. Jun 1 2019;48(3):728–742. doi:10.1093/ije/dyy258
14. Bowden J, Del Greco MF, Minelli C, Davey Smith G, Sheehan N, Thompson J. A framework for the investigation of pleiotropy in two-sample summary data Mendelian randomization. *Stat Med*. May 20 2017;36(11):1783–1802. doi:10.1002/sim.7221
15. Bowden J, Davey Smith G, Burgess S. Mendelian randomization with invalid instruments: effect estimation and bias detection through Egger regression. *Int J Epidemiol*. Apr 2015;44(2):512–25. doi:10.1093/ije/dyv080
16. Burgess S, Thompson SG. Interpreting findings from Mendelian randomization using the MR-Egger method. *Eur J Epidemiol*. May 2017;32(5):377–389. doi:10.1007/s10654-017-0255-x
17. Bowden J, Davey Smith G, Haycock PC, Burgess S. Consistent Estimation in Mendelian Randomization with Some Invalid Instruments Using a Weighted Median Estimator. *Genet Epidemiol*. May 2016;40(4):304–14. doi:10.1002/gepi.21965
18. Mounier N, Kutalik Z. Bias correction for inverse variance weighting Mendelian randomization. *Genet Epidemiol*. Jun 2023;47(4):314–331. doi:10.1002/gepi.22522
19. Sanderson E, Davey Smith G, Windmeijer F, Bowden J. An examination of multivariable Mendelian randomization in the single-sample and two-sample summary data settings. *Int J Epidemiol*. Jun 1 2019;48(3):713–727. doi:10.1093/ije/dyy262
20. Grant AJ, Burgess S. A Bayesian approach to Mendelian randomization using summary statistics in the univariable and multivariable settings with correlated pleiotropy. *Am J Hum Genet*. Jan 4 2024;111(1):165–180. doi:10.1016/j.ajhg.2023.12.002

Supplementary Figures

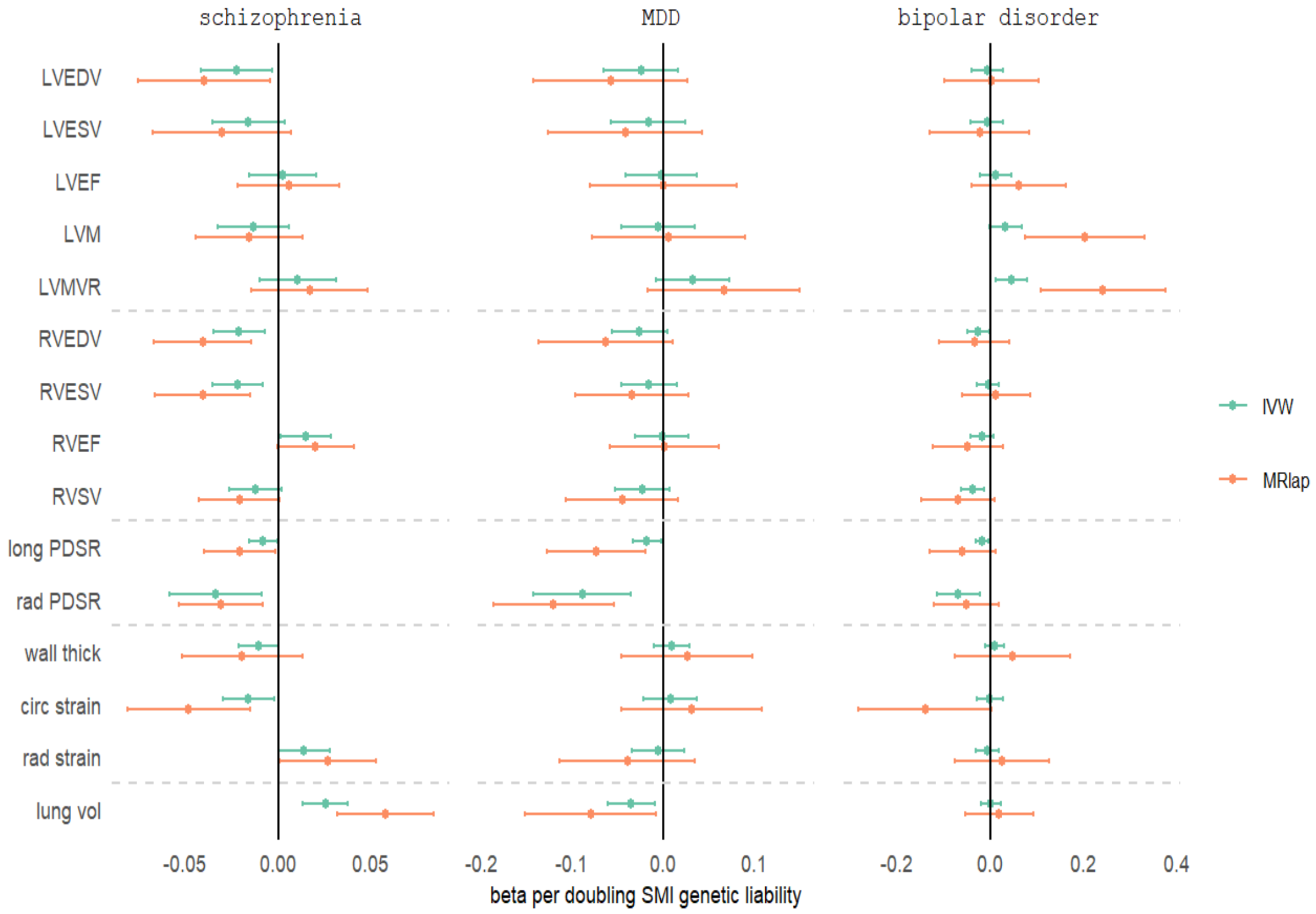

Supplementary Figure 1 Causal effects on cardiopulmonary traits from inverse variance weighted (IVW) estimator and MRlap. Beta coefficients and standard errors were transformed to reflect effects per doubling of genetic liability to severe mental illness (SMI). IVW estimations including all SNPs are represented in green, and MRlap estimations are represented in orange. Error bars represent 95% confidence intervals of the effect sizes. LVEDV: left ventricle end-diastolic volume, RVEDV: right ventricle end-diastolic volume, LVESV: left ventricle end-systolic volume, RVESV: right ventricle end-systolic volume, LVEF: left ventricle ejection fraction, RVEF: right ventricle ejection fraction, LVM: left ventricle mass, LVMVR: left ventricular mass to end-diastolic volume ratio. PDSR: peak diastolic strain rates; long PDSR: longitudinal PDSR, rad PDSR: radial PDSR, wall thick: mean wall thickness; circ strain: mean peak circumferential strain, rad strain: mean peak radial strain, vol: volume; MDD: Major Depressive Disorder.

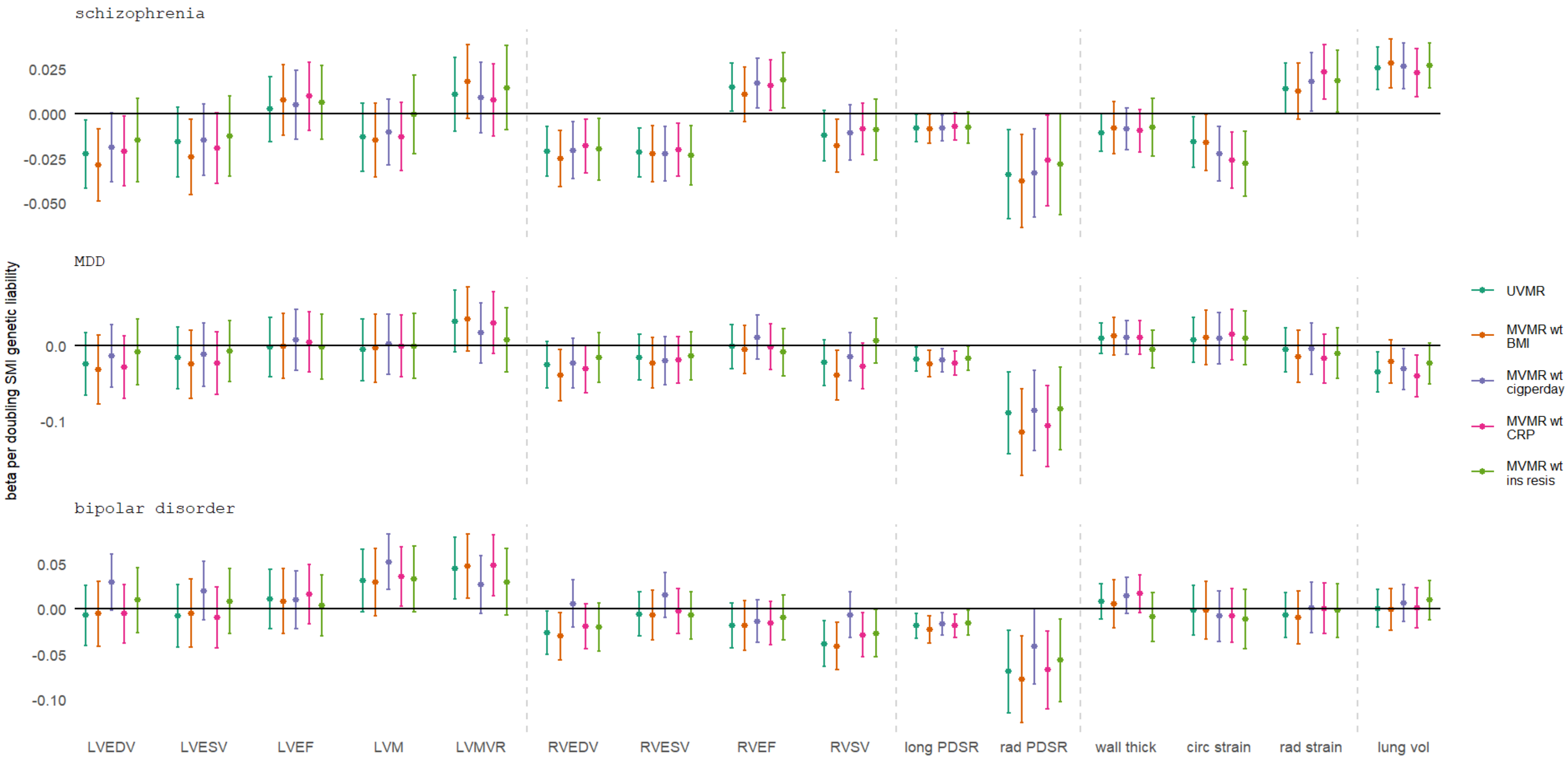

Supplementary Figure 2 Causal effects on cardiopulmonary traits from inverse variance weighted (IVW) estimator and multivariable MR (MVMR) analyses controlling for body mass index (BMI), smoking (cigarette per day), C-reactive protein (CRP), and insulin resistance. Beta coefficients and standard errors were transformed to reflect effects per doubling of genetic liability to severe mental illness (SMI). Univariate (UVMR) IVW estimations are

represented in red, MVMR estimations controlling for BMI are represented in blue, MVMR estimations controlling for smoking are represented in green, MVMR estimations controlling for CRP are represented in purple, and MVMR estimations controlling for insulin resistance are represented in yellow. Error bars represent 95% confidence intervals of the effect sizes. LVEDV: left ventricle end-diastolic volume, RVEDV: right ventricle end-diastolic volume, LVESV: left ventricle end-systolic volume, RVESV: right ventricle end-systolic volume, LVEF: left ventricle ejection fraction, RVEF: right ventricle ejection fraction, LVM: left ventricle mass, LVMVR: eft ventricular mass to end-diastolic volume ratio. PDSR: peak diastolic strain rates; long PDSR: longitudinal PDSR, rad PDSR: radial PDSR, wall thick: mean wall thickness; circ strain: mean peak circumferential strain, rad strain: mean peak radial strain, vol: volume; MDD: Major Depressive Disorder.

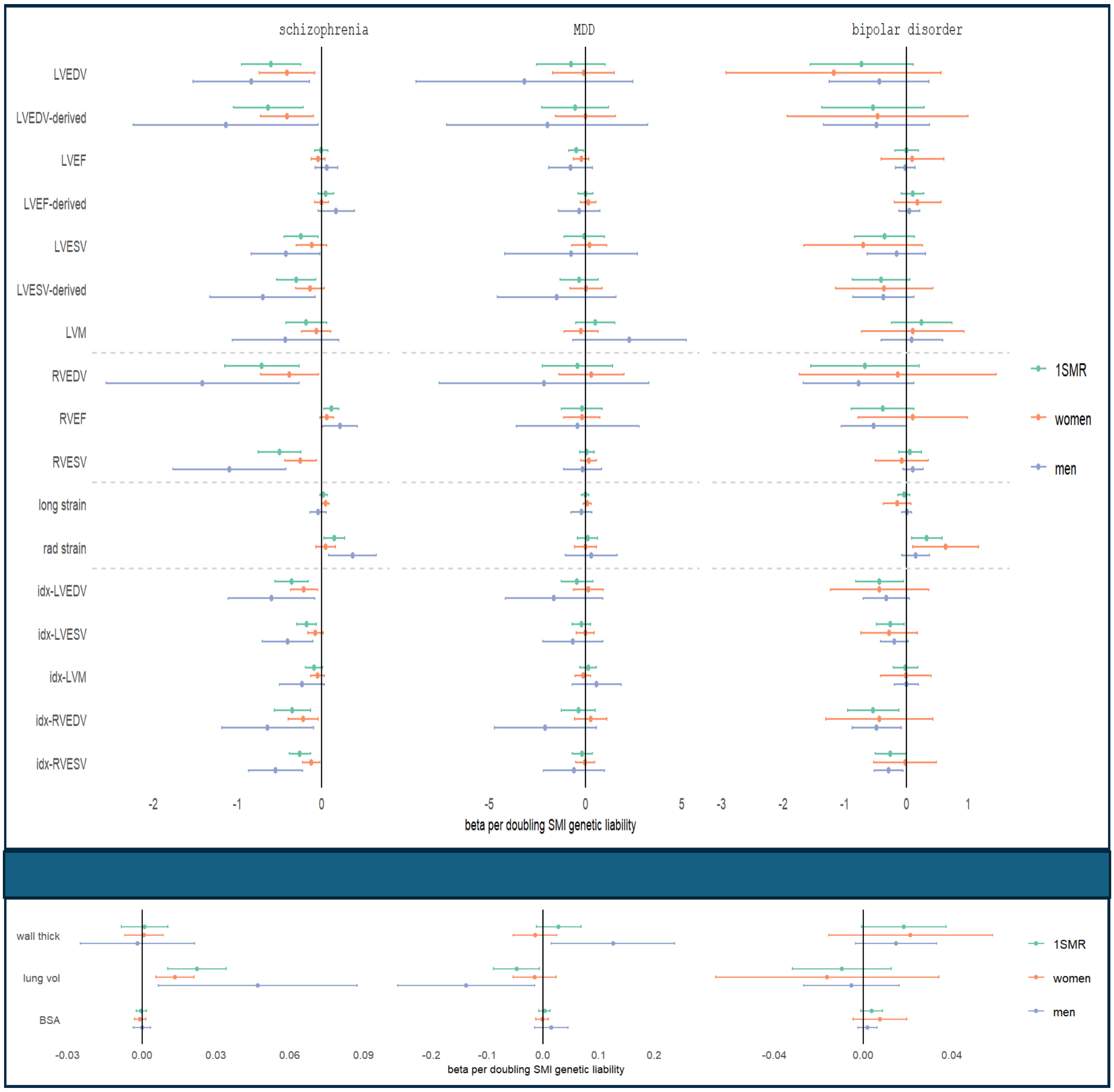

Supplementary Figure 3 Causal effects and sex-specific effects on cardiopulmonary traits from one-sample Mendelian Randomisation (MR). Beta coefficients and standard errors were transformed to reflect effects per doubling of genetic liability to severe mental illness (SMI). One-sample MR estimations are represented in green, estimations in women are represented in orange, and estimations in men are represented in blue. Error bars represent 95% confidence intervals of the effect sizes. LVEDV: left ventricle end-diastolic volume, RVEDV: right ventricle end-diastolic volume, LVESV: left ventricle end-systolic volume, RVESV: right ventricle end-systolic volume, LVEF: left ventricle ejection fraction, RVEF: right ventricle ejection fraction, LVM: left ventricle mass, LVMVR: left ventricular mass to end-diastolic volume ratio. PDSR: peak diastolic strain rates; long PDSR: longitudinal PDSR, rad PDSR: radial PDSR, wall thick: mean wall thickness; circ strain: mean peak circumferential strain, rad strain: mean peak radial strain, vol: volume, BSA: body surface area; SMI: MDD: Major Depressive Disorder. 1SMR: One-sample MR.

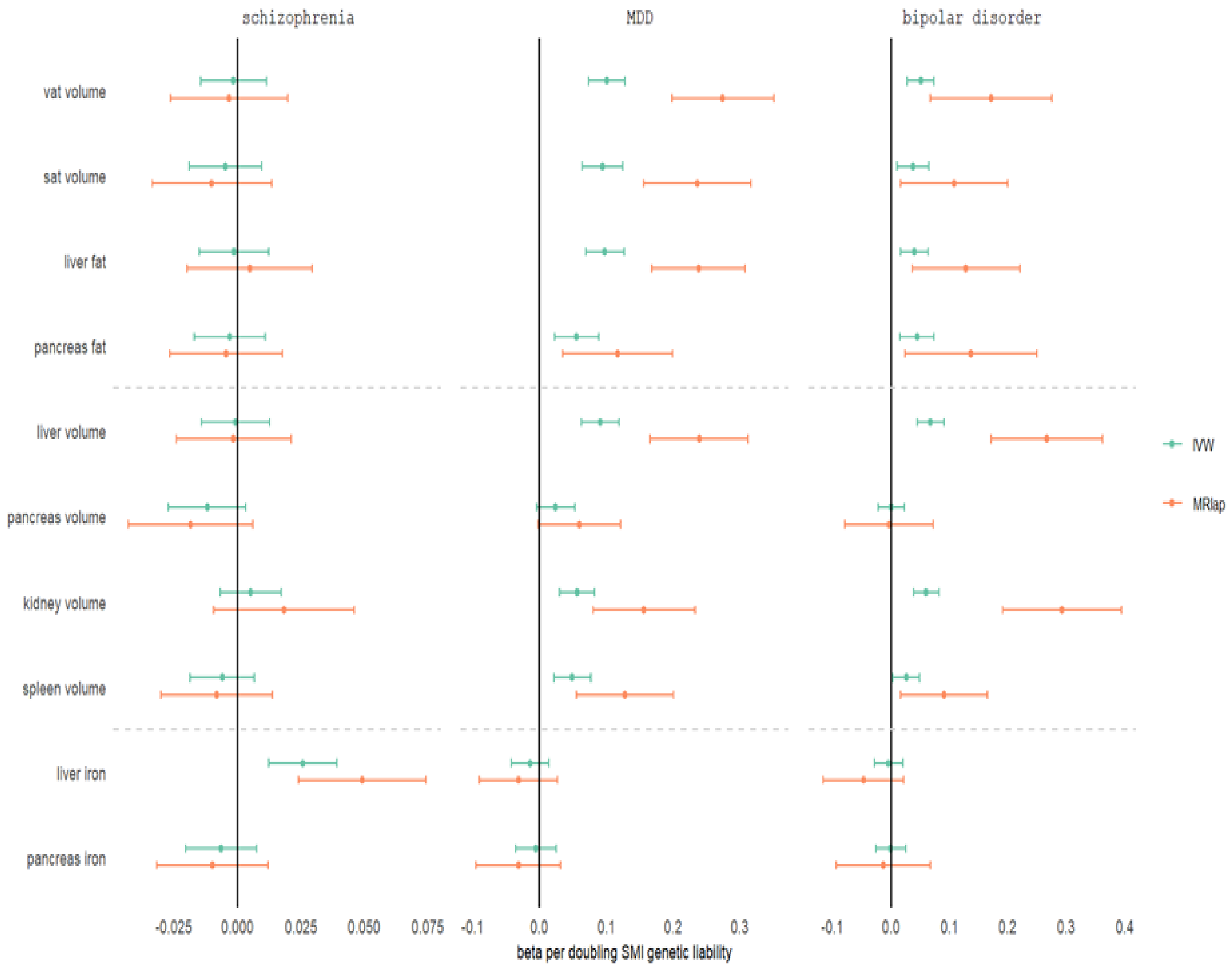

Supplementary Figure 4 Causal effects on abdominal and body composition traits from inverse variance weighted (IVW) estimator and MRlap. Beta coefficients and standard errors were transformed to reflect effects per doubling of genetic liability to severe mental illness (SMI). IVW estimations including all SNPs are represented in green, and MRlap estimations are represented in orange. Error bars represent 95% confidence intervals of the effect sizes. VAT: visceral adipose tissue, SAT: subcutaneous adipose tissue; MDD: Major Depressive Disorder.

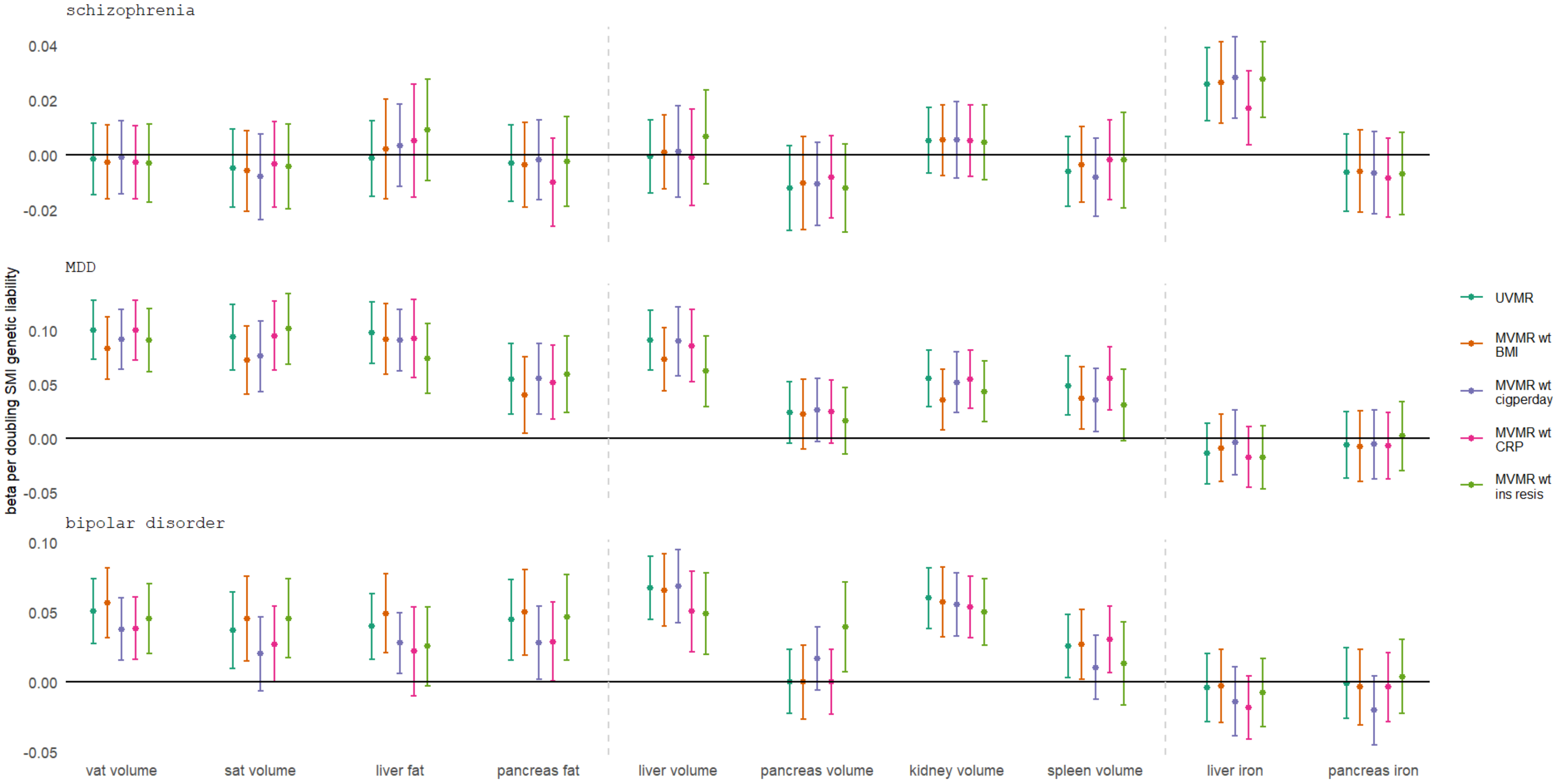

Supplementary Figure 5 Causal effects on abdominal and body composition traits from inverse variance weighted (IVW) estimator and multivariable MR (MVMR) controlling for body mass index (BMI), smoking (cigarette per day), C-reactive protein (CRP), and insulin resistance. Beta coefficients and standard errors were transformed to reflect effects per doubling of genetic liability to severe mental illness (SMI). Univariate (UVMR) IVW

estimations are represented in red, MVMR estimations controlling for BMI are represented in blue, MVMR estimations controlling for smoking are represented in green, MVMR estimations controlling for CRP are represented in purple, and MVMR estimations controlling for insulin resistance are represented in yellow. Error bars represent 95% confidence intervals of the effect sizes. VAT: visceral adipose tissue, SAT: subcutaneous adipose tissue; MDD: Major Depressive Disorder.

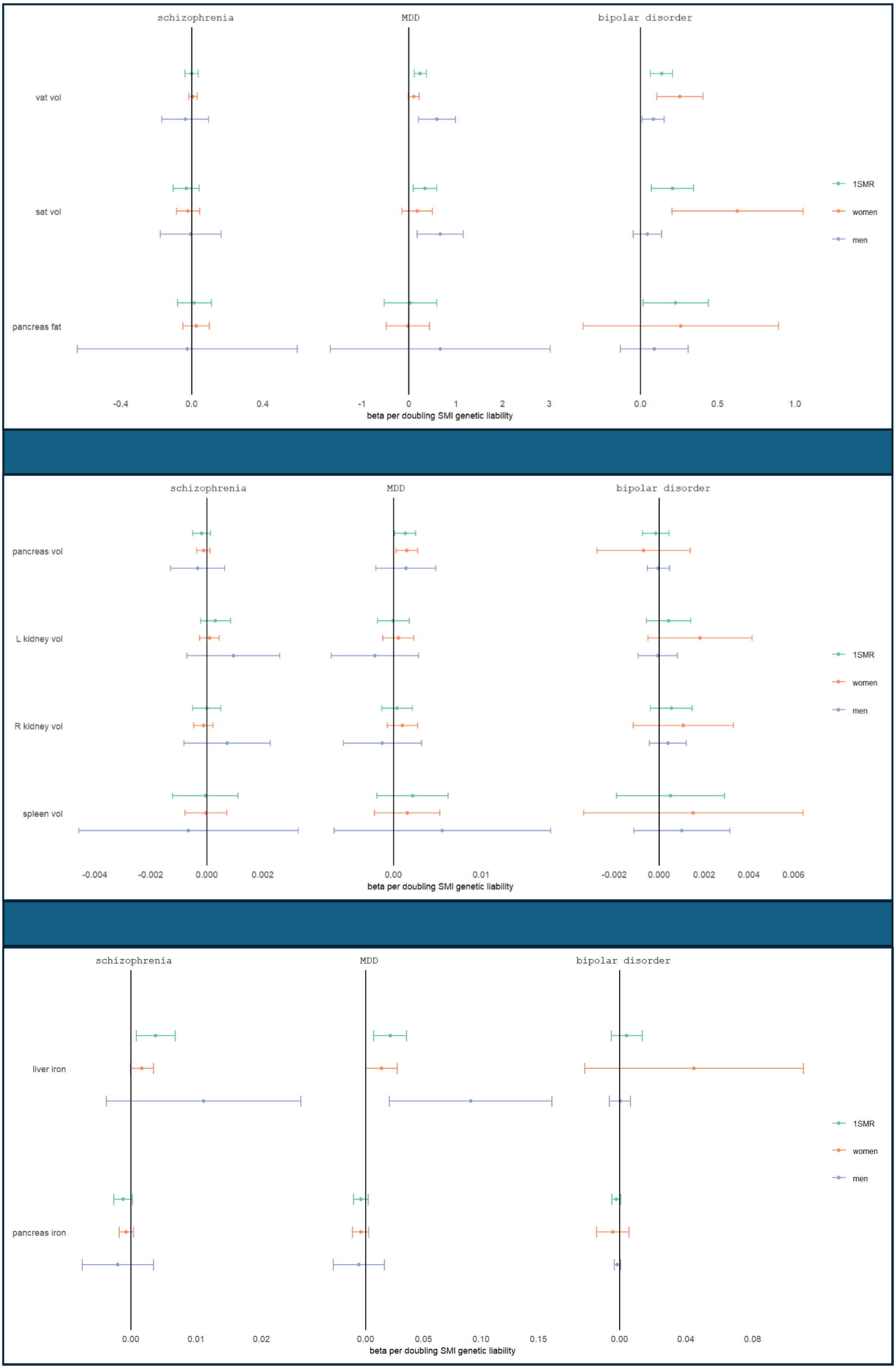
schizophrenia
MDD
bipolar disorder
vat vol
sat vol
pancreas fat
1SMR
women
men
-0.4
0.0
0.4
-1
0
1
2
3
0.0
0.5
1.0
beta per doubling SMI genetic liability
schizophrenia
MDD
bipolar disorder
pancreas vol
L kidney vol
R kidney vol
spleen vol
1SMR
women
men
-0.004
-0.002
0.000
0.002
0.00
0.01
-0.002
0.000
0.002
0.004
0.006
beta per doubling SMI genetic liability
schizophrenia
MDD
bipolar disorder
liver iron
pancreas iron
1SMR
women
men
0.00
0.01
0.02
0.00
0.05
0.10
0.15
0.00
0.04
0.08
beta per doubling SMI genetic liability

Supplementary Figure 6 Causal effects and sex-specific effects on abdominal and body composition traits from one-sample Mendelian Randomisation (MR). Beta coefficients and standard errors were transformed to reflect effects per doubling of genetic liability to severe mental illness (SMI). One-sample MR estimations are represented in green, estimations in women are represented in orange, and estimations in men are represented in blue. Error bars represent 95% confidence intervals of the effect sizes. VAT: visceral adipose tissue, SAT: subcutaneous adipose tissue; MDD: Major Depressive Disorder.
